# Capsaicin Analogue Supplementation Does Not Improve 10 km Running Time-Trial Performance in Male Amateur Athletes: A Randomized, Crossover, Double-Blind and Placebo-Controlled Study

**DOI:** 10.3390/nu13010034

**Published:** 2020-12-24

**Authors:** Ana Elisa von Ah Morano, Camila S. Padilha, Vinicius Aparecido Matos Soares, Fabiana Andrade Machado, Peter Hofmann, Fabrício E. Rossi, Fábio Santos Lira

**Affiliations:** 1 Exercise and Immunometabolism Research Group, Department of Physical Education, São Paulo State University (UNESP), Presidente Prudente 19060-900, Brazil; anaelisavhm@yahoo.com.br (A.E.v.A.M.); camilapersonal@yahoo.com.br (C.S.P.); vihhvinicius@hotmail.com (V.A.M.S.); fabriciorossi@ufpi.edu.br (F.E.R.); 2 Post-Graduate Program of Physiological Sciences, Department of Physiological Sciences, State University of Maringá, Maringá 87020-900, Brazil; famachado_uem@hotmail.com; 3 Associate Post-Graduate Program in Physical Education UEM/UEL, Department of Physical Education, State University of Maringá, Maringá 87020-900, Brazil; 4 Exercise Physiology, Training & Training Therapy Research Group, Institute of Human Movement Science, Sport and Health, University of Graz, 8036 Graz, Austria; peter.hofmann@uni-graz.at; 5 Immunometabolism of Skeletal Muscle and Exercise Research Group, Federal University of Piauí (UFPI), Teresina PI 64049-550, Brazil

**Keywords:** pepper, running races, ergogenic aid

## Abstract

Background: To investigate the acute effects of a capsaicin analogue supplement on 10 km time-trial performance and physiological responses in amateur athletes. Methods: Twenty-one participants (age = 29.3 ± 5.5 years, weight 74.2 ± 11.3 kg, height 176.0 ± 0.0 cm, fat mass 12.7 ± 3.8%, $\dot{V}$O_2max_ 62.7 ± 8.4 mL·k^−1^·min^−1^), completed two randomized, double-blind trials: capsaicin analogue condition (Capsiate (CAP) = 24 mg) or a placebo (PLA) condition. The participants consumed two doses of 12 mg of CAP or PLA capsule 45 min before and immediately at the start of each trial. The time required to complete 10 km, lactate concentration, maximum heart rate (HR_peak_), and rating of perceived exertion (RPE) were recorded. Results: The 10 km time-trial performance (CAP = 45.07 ± 6.41 min vs. PLA = 45.13 ± 6.73, *p* = 0.828) was not statistically significantly different between conditions. No statistically significant differences between conditions were detected for lactate concentration (*p* = 0.507), HR_peak_ (*p* = 0.897) and RPE (*p* = 0.517). Conclusion: Two doses of a 12 mg Capsaicin analogue supplement did not improve performance and physiological responses in a 10 km running time-trial in amateur athletes.

## 1. Introduction

Long-distance running is one of the most common individual sports modalities and has become even more popular in the last decade [1]. Long-distance running promotes several systemic and muscle adaptations [2], increases maximal oxygen uptake ($\dot{V}$O_2max_) [3] and time to exhaustion [4]. The oxidative system demand of long-distance runners is about 3-fold higher compared with physically active people [5,6], and a relative increase of type IIa at expense of type IIb, results in the ability to increase fat metabolism [7]. Moreover, long-distance running provokes a calcium imbalance, which results in inefficiency of muscular contraction [8]. Therefore, different ergogenic and thermogenic compounds were strategically used to optimize performance in middle- and long-distance running [9].

Capsiate (CAP) is an analogue of capsaicin, belonging to an 8-methyl-N-vanillyl-trans-6-nonenamide natural phytochemical compound found primarily in red peppers [10] and also in the analogue of a sweet pepper species (CH-19 sweet) which has the same molecular structure as capsaicin, except for the substitution of NH for O in the alkyl chain that alters the pungency factor [11]. This phytochemical has been used as a nutritional strategy to improve performance in different intensities and exercises distances [12,13].

The dose of capsaicin is not well established in humans. Previously, studies from our research group have used 12 mg of CAP 45 min before the exercise investigating different type of protocols (400, 1500, and 3000 m and in high-intensity intermittent exercise) in physically active men [12,13,14]. Additionally, improvements in combined exercise protocols, such as 5 km running plus strength exercise were detected applying 24 mg of CAP in two doses of 12 mg with 45 min interval between doses [15]. On the other hand, another study showed improved endurance performance using 150 mg CAP administered 60 min before a 30 min aerobic cycle ergometer exercise bout, demonstrating higher fat oxidation under CAP conditions compared to PLA [16]. Some time ago, Szallasi and Blumberg (1999) [17] reported that dosages above 33 mg per day of capsaicinoids induced gastric discomfort in humans. Based on this report, a pilot study identified that lower dosages (12 or 24 mg) can improve performance without those side effects in humans (de Freitas et al. 2018, 2019a, 2019b; Costa et al. 2020) although the optimal dose–response of CAP in 10 km running is still unknown. Peak concentration of CAP is suggested to occur after about 45 min after ingestion and bioavailability is approximately 25 min, with increased plasma values for up to 105 min [18,19].

In skeletal muscle, CAP phosphorylates the transient vanilloid-1 receptor (TRPV1), which provokes the release of available calcium from the sarcoplasmic reticulum, optimizing myosin–muscle actin interaction, improving the processes of force generation and optimizing the efficiency in depleting intramuscular triglycerides. These effects preserve muscle glycogen, and consequently increases time to exhaustion [20]. Moreover, TRPV1 is activated in the putative pain neural circuit [21] increasing fat metabolism, at least in part, due to β-adrenergic stimulation induced by CAP supplementation. Therefore, the CAP ingestion may increase fat metabolism by both stimulating lipolysis and inhibiting lipogenesis [22], which could potentiate longer distance performance.

Regarding the potential ergogenic effect of CAP, previous studies from our research group demonstrated that 12 mg of acute CAP supplementation was effective to improve 1500 m running time-trial performance with a lower rate of perceived exertion (RPE) [13]. Recently, an investigation about running performance and CAP verified the effect of acute CAP supplementation on short (400 m) and moderate distance (3000 m) running time-trial performance in physically active men but they did not find differences in heart rate (HR) and RPE [14]. On the other hand, [23] observed that the acute CAP supplementation did not increase time to exhaustion during high-intensity continuous exercise (90% $\dot{V}$O_2__peak_) nor did it alter physiological responses in runners. Despite several studies that have investigated the potential effect of CAP on endurance performance is primarily in rodents [24], no information is available for short- and middle-distance [13,22] or high-intensity intermittent exercise in humans [12,25] beyond a certain distance regarding the effects of the CAP supplementation, such as in the 10 km time-trial. Furthermore, previous studies demonstrating the benefits of CAP on performance were conducted in physically active men; therefore, there is a lack of research investigating the effect in experienced runners.

Thus, the objective of this study was to verify the acute effect of CAP supplementation on 10 km running time-trial performance and physiological responses in amateur athletes. We hypothesize that acute CAP supplementation may be a viable strategy in improving long-distance performance (10 km) with lower lactate concentration, HR_peak_, and RPE.

## 2. Materials and Methods

### 2.1. Experimental Design

**Figure 1:**
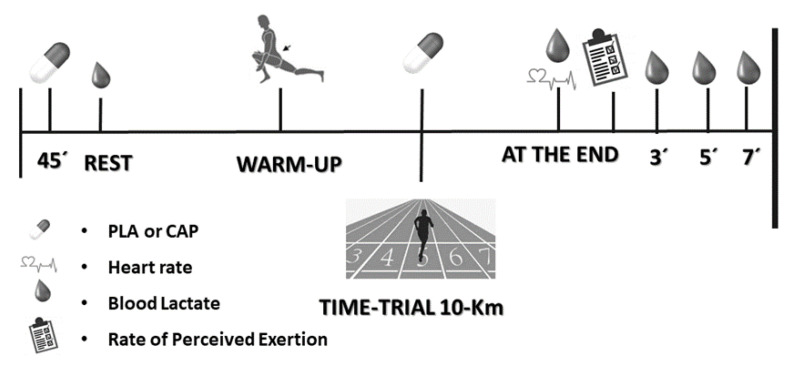
Experimental design. PLA = placebo. CAP = capsaicin.

This study is a randomized, crossover, double-blind and placebo-controlled study registered in “Registro Brasileiro de ensaios clinicos” (ID: RBR-2qfvyfv). The experimental trials were conducted in the morning (6 to 9 a.m.) under similar weather conditions (relative humidity: 60–90%; wind: 5–29 km.h^−1^; temperature: 19–26 °C; altitude: 475 m) to minimize the chronobiological variance. The participants were instructed about the protocol and were requested to maintain their training and nutritional habits during the study. The participants visited the track for two 10 km running tests under CAP or PLA conditions separated by one week (Figure 1). The trials were performed on a 400 m official outdoor track, between March to May (in total, it took eight weeks to have all data collected completed).

### 2.2. Participants

**Figure 2:**
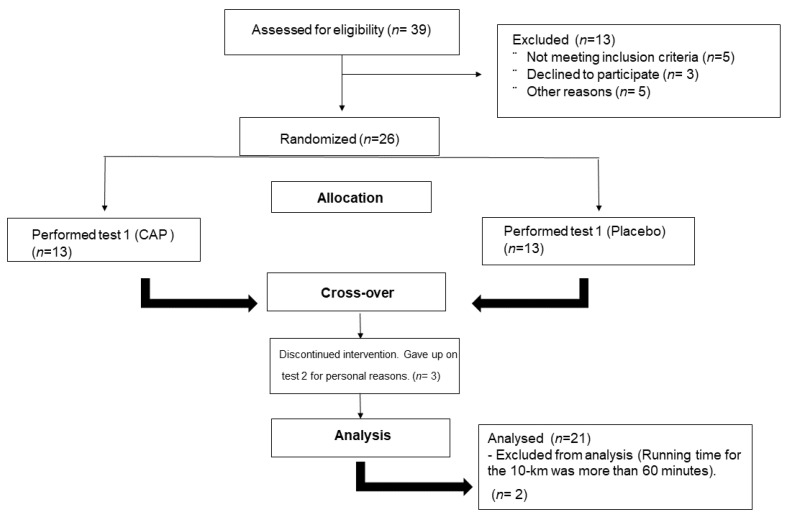
Flow Diagram.

Twenty-one male, amateur athletes were included in this study (Figure 2). The general characteristics of participants are presented in Table 1. All participants had at least one year of experience in running and were required to be 18–35 years old. Study exclusion criteria were any cardiovascular, muscle or joint contraindications to exercise as well as use of any ergogenic supplementation. Subjects had a 10 km running time between 34.3 and 57.4 min (mean velocity between 10.3 and 17.3 km.h^−1^ representing ≅45.7 and 76.4% of the world record) [26].

They were advised to abstain from chili peppers or other spicy foods, caffeinated, supplements or ergogenic substances, energy drinks (which contain any kind of stimulant ingredient) or alcoholic beverages as well as strenuous physical exercise within 24 h prior to testing. Subjects were requested to maintain their regular food intake, and to maintain the same physical exercise regimen 48 h prior to testing. Additionally, they were instructed to consume breakfast at home as usual, before each experimental trial. Overall energy consumption (kilocalories) and macronutrient intake was calculated from the Brazilian food composition table (TACO) to ensure a similar intake in both experimental trials. The Research Ethics Committee (protocol number 3,654,560/2019) approved the study protocol which was conducted according to the 2013 Revision of the Declaration of Helsinki. The subjects signed a consent form and were informed about the purpose of the study and possible risks.

### 2.3. Supplementation Protocol

A researcher who did not participate in the study (double-blinded) randomized the participants by a random sequence generator a (www.Random.org) in PLA and CAP condition trial. The second trial was performed in a cross-over manner. In both conditions, subjects ingested two capsules of 12 mg of CAP (24 mg) or PLA according to our previous study that demonstrated benefits of such an application rate [27]. Participants ingested one capsule 45 min before the test and the second one immediately before the start of the 10 km running test. This strategy of CAP supplementation was applied to use peak concentration occurring at about 45 min after intake and a bioavailability of approximately 25 min, to remain the plasma level elevated up to 105 min [18,19]. The product used for this study contained 50% extract from capsicum (*capsicum annuum* L.) from India (Purifarma-Gemini Pharmaceutical Industry Ltd., Anapolis, GO, Brazil) and 50% of maltodextrin. The correction factor in assay calculation was used by Pharma Nostra (Campinas, Brazil) to guarantee 100% of capsinoids (Capsiate) in each capsules of 12 mg. The capsules were identical and without flavor, the PLA capsule content was 50 mg of starch. It was delivered to each subject by an independent person who did not participate in the research team in order to secure double-blinding.

### 2.4. 10 km Time-Trial Running Test

All participants had previous experience in long distance running and were familiar with the track where the tests were carried out. Attempts 1 and 2 were performed without the presence of opponents or another competitor on the track. The trial was preceded by a self-determined warm-up of 10 min, the same in both trials. All participants were encouraged to give their best performance and, in each lap, they were cheered by the research team. Participants freely choose their pacing strategy during the performance and the time in minutes was recorded every 400 m. The overall mean velocity for each trial was calculated by dividing the total distance covered by the total time of the test duration (pace strategy). All subjects had access to mineral water during the run *ad libitum*.

### 2.5. Blood Lactate

Twenty-five microliters of blood were collected from the volunteer’s right ear lobe before, immediately after, and 3, 5 and 7 min after trial. The lactate concentration was determined by electroenzymatic methods using an automated analyzer (YSI 2300 STAT^®^, Yellow Springs, OH, USA). Peak lactate concentration was defined for each participant as the highest post-exercise lactate concentration value [28].

### 2.6. Rating of Perceived Exertion and Heart Rate

Rating of perceived exertion was evaluated by the 6–20 points Borg scale and peak of heart rate (Polar Vantage NV, Electro Oy, Finland) was recorded immediately after the 10 km was completed.

### 2.7. Incremental Test

To determine aerobic fitness, the participants performed a maximal incremental test on the treadmill (Inbramed ATL^®^, Porto Alegre, RS, Brazil) until exhaustion. The maximum oxygen uptake ($\dot{V}$O_2max_) was determined by the latest 30 s mean from the last stage completed in the incremental test. Gas exchanges were measured breath-by-breath using a gas analyzer (Model Quark PFT Ergo—Cosmed^®^—Rome, Italy). Before each test, the gas analyzer was calibrated according to the manufacturer’s recommendations. The participants performed three minutes of warm-up at 8 km·h^−1^. Each stage of the test lasted one minute and the first stage was performed at 9 km·h^−1^, with speed increments of 1 km·h^−1^ per stage until voluntary exhaustion by the participants [29]. The maximal velocity (V_max_) from the incremental was assumed as the highest velocity in the stage completed before exhaustion. Later, the mean from the Vmax was compared to the mean velocity of the 10 km running time-trial test (Table 2).

### 2.8. Body Composition and Anthropometry

All the procedures were performed by the same person in an acclimatized room. Body mass was measured on an electronic scale (Filizzola PL 150, Filizzola^®^ Ltd.a, São Paulo, Brazil). Body height was measured with a wall-mounted stadiometer (Sanny^®^, São Paulo, Brazil). Fat-free mass and fat mass were estimated by bioelectrical impedance following the procedure of the manufacturer (Bia Analyzer TM^®^, The Nutritional Solutions Corporation, Harrisville, MI, USA).

### 2.9. Statistical Analysis

The sample size of this study was calculated according to previous studies from our research group that verified the improvement of running time in CAP conditions [13,14]. An effect size of 0.60 as well as β − 1 = 0.85 were considered for the power analysis using G*Power software. A sample size of *n* = 21 was calculated to sufficient to detect the prescribed effects within this study protocol.

Data were analyzed using Statistical Package for the Social Sciences (SPSS^®^ v.24, Inc., Chicago, IL, USA). Data normality was verified using the Shapiro–Wilk test. Data are reported as means and ± standard deviation (SD). The intraclass correlation coefficient (ICC) and coefficient of variation (CV) test was applied to verify the reliability between trial 1 and 2 (blinded) and PLA and CAP trial. A paired *t* test was used to compare both conditions (CAP and PLA). Two-way analysis of variance (ANOVA) for repeated measures was used for both within-group and between-group comparisons. When an F-ratio was significant, Tukey’s HSD post hoc test was used to identify significant differences. If the sphericity of variances was violated, as indicated by Mauchly’s test, the analyses were adjusted with a Greenhouse–Geisser correction. Statistical significance was set at *p* < 0.05.

## 3. Results

Participant’s general characteristics are presented in Table 1. There was no difference between the trials for total kilocalorie and total macronutrient intake 24 h before exercise for each condition (*p* > 0.05) Table 2.

The first and the second trial and both conditions (PLA vs. CAP) were compared in order to identify the influence of learning effects. No significant differences between the first and the second attempt of the 10 km runs were found (*p* > 0.05) (Table 3).

**Figure 3:**
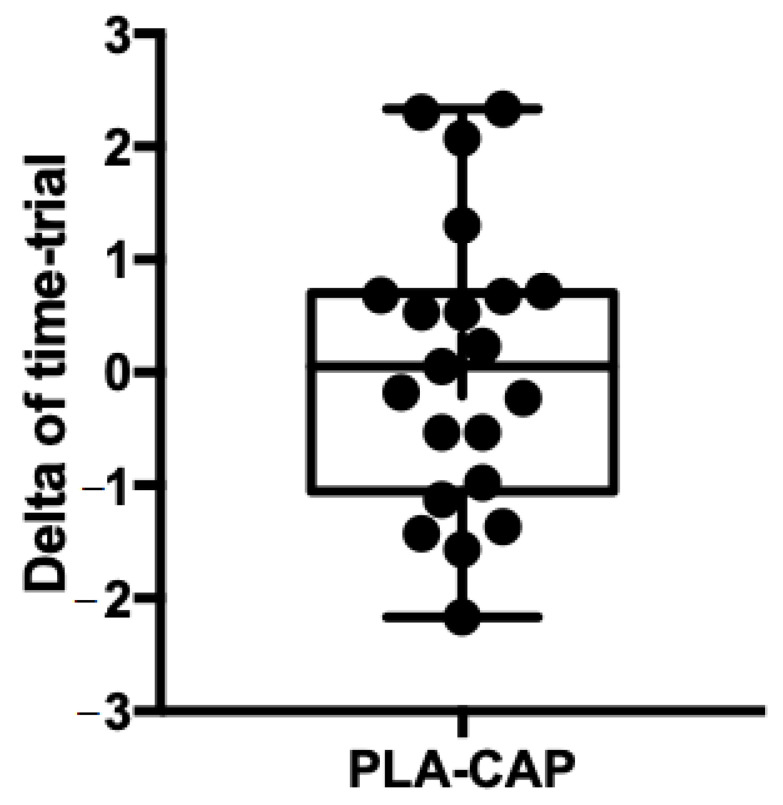
Delta individuals’ values of time-trial (PLA-CAP).

**Figure 4:**
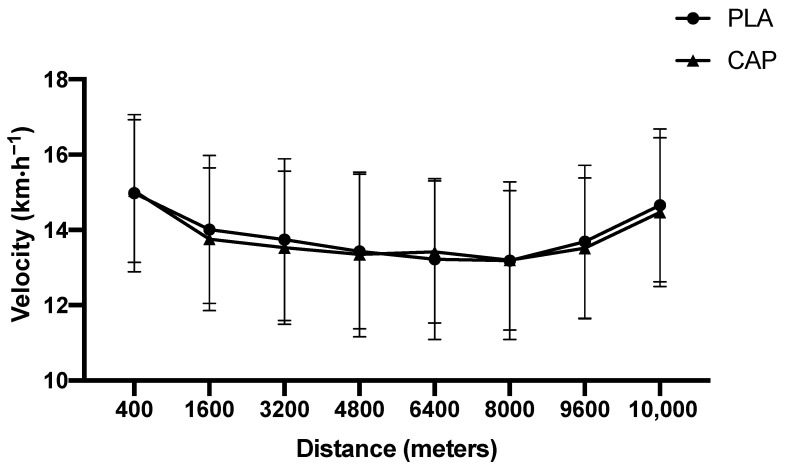
Mean running velocity during a 10 km running time-trial.

Comparing the effects of CAP versus PLA conditions on 10 km running time did not reveal any significant differences between conditions. Additionally, no statistically significant differences were detected for lactate concentration, HR_peak_ and RPE between conditions. The delta of individual values showed those participants who improved or worsened their time-trial between PLA and CAP conditions (Figure 3). There was no interaction main effect (PLA vs. CAP condition * distance) (*p* = 0.996), the participants presented similar mean velocity changes along distances in both condition (PLA and CAP) (Figure 4).

## 4. Discussion

According to our knowledge, this is the first study to investigate the acute effect of CAP supplementation on 10 km time-trial performance in male amateur athletes. Our main findings regarding CAP supplementation effects on performance are in contrast to previous studies from our research group investigating these effects for shorter distances. The study conducted by de Freitas et al. [13] demonstrated that CAP supplementation improved 1500 m time-trial performance with lower RPE but no changes of lactate concentrations in physically active adults. In addition, acute effects of CAP supplementation were verified to improve performance of short (400 m) and moderate distance (3000 m) running time-trials without changes of HR_peak_ and RPE in physically active men [14]. Therefore, we refute our initial hypothesis that CAP supplementation has an ergogenic effect improving performance and physiological responses in athletes performing a 10 km time-trial.

Interestingly, in these previous studies, CAP reduced 1500 m running time-trial performance by 1.35%, decreased running time by 1.04% in 400 m running time-trial and 2.32% in a 3000 m running time-trial in physically active men [14]. From these data, we might expect an even larger effect on 10 km running time-trial performance, as the increase in performance was suggested to be dependent on distance. However, in the current study, there were no significant effects of acute CAP on performance and physiological responses during a 10 km running time-trial in male amateur athletes. Indeed, these findings suggest that the duration of effort (short, middle and longer distance), as well as the training status of subjects, may be important factors with regard to the potential ergogenic benefits of CAP supplementation on exercise performance. Our previous studies [12,13,14] demonstrated benefits of acute CAP application in physically active subjects but not in the current study with male amateur athletes.

In contrast, several animal studies have shown that acute capsaicin supplementation was efficient to improve endurance capacity (exercises protocols between 30–60 min of moderate aerobic exercise) [24]. The physiological mechanisms explaining these effects have been related in part to an increase in hepatic glycogen content, being considered an important mechanism for energy homeostasis [24]. Additionally, the activation of TRPV1 due to the capsaicin supplementation and improvements of energy metabolism by increasing lipolysis and saving and maintaining muscle glycogen reserves were suggested [24]. Kazuya et al. 2014 [30] demonstrated that 100 mg of CAP improved the control exerted from adenosine diphosphate (ADP) on mitochondrial respiration, facilitating the potent CAP-induced activation of mitochondrial decoupling processes, which leads to the dissipation of the proton gradient generated throughout the respiratory chain. Additionally, oxidative ATP contribution in electrostimulation-induced muscle was enhanced and ATP cost of twitch force generation was reduced. Acute CAP supplementation also enhanced the twitch force-generating capacity. It is, therefore, suggested that acute CAP supplementation may alleviate fatigue during resistance exercises [31]. Although these studies showed evidence for CAP supplementation effects on endurance performance, we did not find any improvements in our 10 km running time-trial.

All subjects were encouraged to run as fast as possible, giving their best for an excellent performance; however, it was not an official race and, therefore, motivation influences cannot be ruled out. A few key limitations must be discussed in this study. First, there was no familiarization or practice session before having the participants complete the experimental trials. Due to the wide array of performance ability identified in this study participants, the lack of a familiarization trial likely created more variability between outcomes that confounded any ability to identify the impact of supplementation. Additionally, the performance ability of our cohort study was quite variable, with some participants running nearly 25 min faster than others on the same test. This likely led to situations where changes in performance were more related to a relative lack of running background when compared to others. It is important to highlight that our findings cannot be related to female athletes due to the possible hormonal influences and different phases of menstrual cycle which might interfere with performance. Moreover, several related markers such as potential hydrogenic (pH), inorganic phosphate, glycogen depletion, calcium release or catecholamine response have not been measured which need to be included in subsequent studies to increase the understanding about CAP supplementation and endurance running performance.

## 5. Conclusions

Our study failed to show an ergogenic effect of acute CAP ingestion on time to exhaustion, blood lactate concentration, heart rate response or perceived exertion during a 10 km running time-trial in male amateur athletes at the given application rate. Being the first to investigate the effects of acute CAP supplementation on 10 km running in humans highlights the need to investigate variable doses of CAP supplementation on the activation of the TRPV1 receptor and the subsequent effects on human metabolism in endurance exercise in more detail.

## Figures and Tables

**Table 1:** General characteristics of participants.

Variables.	(*N* = 21)
Age (years)	29.3 ± 5.4
Weight (kg)	74.2 ± 11.3
Height (cm)	176.0 ± 0.0
Fat mass (%)	12.7 ± 3.8
Fat Free Mass (kg)	64.3 ± 7.2
$\dot{V}$O_2max_ (mL·k^−1^·min^−1^)	62.7 ± 8.4

Note: Data are shown in mean ± SD. $\dot{V}$O_2max_ = maximal oxygen uptake.

**Table 2:** Dietary intake and macronutrient distribution 24 h before each trial.

	PLA	CAP	*p*-Value
Total intake (kcal)	1.791± 661	1. 853 ± 476	0.322
Protein (g)	97.2 ± 41.7	107.2 ± 47.7	0.221
Carbohydrate (g)	213.6 ± 97.4	206.6 ± 69.3	0.9
Lipids (g)	61.0 ± 28.4	66.9 ± 26.0	0.112
Total intake (kcal·kg^−1^)	24.8 ± 9.2	21.8 ± 10.9	0.357
Protein (g·kg^−1^)	1.3 ± 0.5	1.2 ± 0.7	0.669
Carbohydrate (g·kg^−1^)	3.0 ± 1.4	2.4 ± 1.3	0.219
Lipids (g·kg^−1^)	0.8 ± 0.3	0.7 ± 0.4	0.66

Note: Data are shown in mean ± SD. PLA = placebo. CAP = capsaicin analogue *p* < 0.05 compared to placebo (PLA) condition.

**Table 3:** Performance and physiological response in 10 km running test at capsiate (CAP) and placebo conditions (*n* = 21).

	Trial 1 (Blinded)	Trial 2 (Blinded)	ICC	CV %	*p*-Value	PLA	CAP	ICC	CV %	*p*-Value
Time 10 km (min)	45.13 ± 0.004	45.00 ± 0.004	0.98	1.61	0.429	45.08 ± 0.004	45.04 ± 0.004	0.99	1.62	0.828
Mean velocity (km·h^−1^)	13.5 ± 1.8	13.6 ± 1.9	0.98	2.66	0.294	13.5 ± 1.9	13.5 ± 1.8	0.99	1.96	0.707
Vmax relative to incremental test (%)	76.1 ± 100	76.6 ± 103.2	0.9	6.13	-	76.5 ± 104.2	76.2 ± 98.9	0.97	2.73	-
HR_peak_ (bpm)	181 ± 12.9	181 ± 12.1	0.62	6.41	0. 798	181 ± 11.2	180 ± 13.5	0.9	6.53	0.942
RPE_peak_ (6–20-point BORG scale)	17 ± 2.0	17 ± 2.3	0.88	6.01	0.55	17 ± 2.3	17 ± 2.0	0.52	10.84	0.55
[La^−^] rest (mmol·L^−1^)	0.8 ± 0.3	0.9 ± 0.5	0.82	8.42	0.457	0.9 ± 0.4	0.8 ± 0.4	0.45	16.9	0.507
Peak [La^−^] (mmol·L^−1^)	5.0 ± 1.6	5.5 ± 1.9	0.94	7.45	0.219	5.2 ± 1.8	5.2 ± 1.8	0.7	5.1	0.95

Note: Data are shown in mean ± SD. HR_peak_ = Heart Rate; RPE = rate of perceived exertion (6–20-point BORG scale); Lac = lactate concentration; ICC = intraclass coefficient of correlation; CV = coefficient of variation; *p* < 0.05 compared to PLA condition.

## Data Availability

The data presented in this study are available on request from the corresponding author. The data are not publicly available due to privacy.
